# Therapeutic Electrical Stimulation Using Belt Electrodes and Nutritional Management in a Patient With Rheumatoid Arthritis and Sarcopenia: A Case Report

**DOI:** 10.7759/cureus.88125

**Published:** 2025-07-16

**Authors:** Norikazu Hishikawa, Shogo Toyama, Koshiro Sawada, Motonori Kubo, Suzuyo Ohashi, Yasuo Mikami

**Affiliations:** 1 Department of Rehabilitation Medicine, Graduate School of Medical Science, Kyoto Prefectural University of Medicine, Kyoto, JPN; 2 Department of Orthopaedics, Graduate School of Medical Science, Kyoto Prefectural University of Medicine, Kyoto, JPN; 3 Department of Rehabilitation, University Hospital, Kyoto Prefectural University of Medicine, Kyoto, JPN; 4 Department of Development of Multidisciplinary Promotion for Physical Activity, Kyoto Prefectural University of Medicine, Kyoto, JPN

**Keywords:** muscle mass, nutritional management, rheumatoid arthritis, sarcopenia, therapeutic electrical stimulation

## Abstract

Patients with sarcopenia are recommended to undergo rehabilitation treatment consisting of resistance training and nutritional management. However, resistance training in patients with rheumatoid arthritis (RA) remains controversial because it may exacerbate joint impairments. Wide-area, low-pain therapeutic electrical stimulation (TES) using belt electrodes is a novel method that induces whole-muscle contractions in the lower limbs and has been increasingly adopted in clinical settings. This report describes a patient with concurrent RA and sarcopenia who underwent combined TES and nutritional management to assess its feasibility and efficacy. The patient was a 73-year-old woman who had been diagnosed with RA over one year ago, had initiated medication, and had low disease activity. As the patient had both RA and sarcopenia, a rehabilitation treatment combining novel TES and nutritional management was implemented over a six-month period. The patient underwent TES for 20 minutes once or twice a week as an outpatient. In addition, she was instructed to drink one nutritional supplemental drink containing the essential amino acid leucine daily at home. No joint-related adverse events occurred during the rehabilitation treatment, and the patient consumed the nutritional supplemental drink with high adherence. After six months, the patient’s muscle mass and strength had increased and physical performance had improved, suggesting that combined TES and nutritional management may be a feasible, safe, and effective strategy for treating sarcopenia in patients with RA. Many patients with RA only receive limited outpatient rehabilitation treatment. Our program combining TES and nutritional management as an alternative to resistance training may represent an important strategy to treat sarcopenia in patients with RA.

## Introduction

The introduction of methotrexate, biologics, and Janus kinase inhibitors has markedly advanced the treatment of rheumatoid arthritis (RA) and enabled many patients to achieve clinical and functional remission [1]. However, patients with RA still have a high risk of joint impairment (i.e., pain and deformity) due to ageing and prolonged disease duration [2]. In addition, the effects of the inflammatory cytokines involved in the chronic inflammatory status of patients with RA easily lead to sarcopenia, which is characterized by a decrease in skeletal muscle mass. Therefore, patients with RA have a very high prevalence of sarcopenia [3] and many need to be started on additional supplementary non-drug treatments, such as rehabilitation treatment [4].

Management guidelines for sarcopenia recommend rehabilitation treatment interventions combining resistance training and nutritional intervention focused on the intake of essential amino acids [5]. However, therapeutic exercise such as resistance training may not be feasible for patients with concurrent RA and sarcopenia who have very low exercise capacity, as it induces stress on the joints and poses a risk for the progression of joint destruction and worsening of pain [6]. Therefore, it is important to select alternatives to resistance training that place less stress on the joints of patients with RA.

Therapeutic electrical stimulation (TES) is a rehabilitation treatment method that induces forced muscle contraction by applying electrical stimulation to skeletal muscles. TES is often used as an alternative for people who have difficulty with therapeutic exercise. However, the conventional TES devices have a limited range of skeletal muscle coverage and cause such severe pain that it is often difficult to achieve sufficient stimulation intensity. Compared with conventional TES, the recently developed TES system using belt electrodes has a larger electrode area and skin contact area, which distributes the current density to significantly reduce the severity of pain during stimulation and induces whole-muscle contraction in the lower limbs [7]. This TES using belt electrodes may safely increase the muscle mass in patients with concurrent RA and sarcopenia; however, evidence supporting this potential outcome remains limited.

This case report aimed to examine the effects of a six-month rehabilitation treatment that combines TES using belt electrodes and nutritional management in a patient with concurrent RA and sarcopenia.

## Case presentation

Patient information

The patient was a 73-year-old woman (height: 154.0 cm, weight: 46.5 kg, body mass index: 19.6 kg/m^2^) who was diagnosed with RA over one year ago and was continuing drug treatment initiated by a rheumatologist in accordance with clinical guidelines. Her drug regimen comprised only iguratimod (50 g/day) as a disease-modifying antirheumatic drug. She attended the Department of Rheumatology about once a month as an outpatient. She had no medical history other than RA. The disease activity was 1 for the tender joint count(range: 0-28 count), 1 for the swollen joint count (range: 0-28 count), 1.0 cm for the evaluator global assessment (range: 0-10 cm), 1.8 cm for the patient global assessment (range: 0-10 cm), and 0.3 mg/dL for C-reactive protein (reference range: 0.0-0.3 mg/dL). Furthermore, the Simple Disease Activity Index (SDAI) [8] was low at 5.1 points. The degree of joint destruction was early-stage RA with a Steinbrocker classification of stage I. There was no limitation of range of motion. The patient had a hand grip strength of 22.1 kg, a 6-m gait speed of 0.5 m/sec, and skeletal muscle mass index (SMI) of 5.3 kg/m^2^, assessed via the bioelectrical impedance analysismethod [9] using a body composition analyzer (InBody S10, InBody Japan Inc., Japan). She was diagnosed with sarcopenia (low SMI plus low physical performance) based on the criteria of the Asian Working Group for Sarcopenia 2019 [10]. In accordance with the Global Leadership Initiative on Malnutrition criteria, malnutrition is diagnosed based on the presence of at least one phenotypic criterion (non-volitional weight loss, low body mass index, or reduced muscle mass) and one etiologic criterion (reduced food intake or assimilation, and inflammation or disease burden) [11]. Therefore, our patient was diagnosed with malnutrition because she had a reduced muscle mass and inflammation. As the low muscle mass were affecting her low physical performance, we proposed rehabilitation treatment to increase the muscle mass of the lower limbs. 

Therapeutic intervention

**Figure 1:**
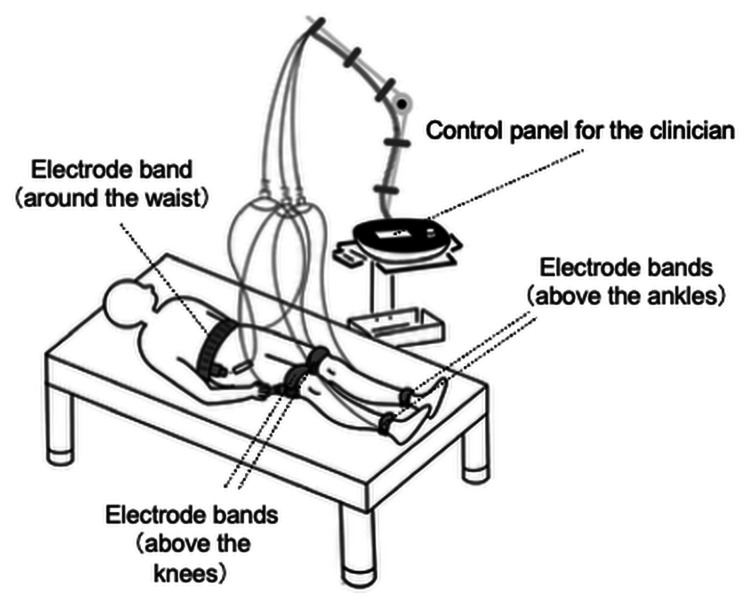
Configuration of the TES system using belt electrodes The belt electrodes were attached at five locations: around the patient’s waist, above both knees, and above both ankles. Electrical stimulation can be applied simultaneously to muscle groups in both lower limbs (e.g., quadriceps femoris, hamstrings, gastrocnemius, soleus, tibialis anterior, and peroneal muscles), with the current intensity adjusted separately for the thighs and lower legs. TES, therapeutic electrical stimulation Image credit: Norikazu Hishikawa

The patient received outpatient rehabilitation treatment performed by a physical therapist for 40 minutes once or twice a week for a six-month period. The rehabilitation program consisted of lower limb stretching, TES using belt electrodes, and gait training. TES using belt electrodes was administered for 20 minutes each time using a General Therapeutic Electrical Stimulator (G-TES; Homer Ion Laboratory Co., Ltd., Tokyo, Japan). As shown in Figure 1, this device was applied using five dedicated silicon-rubber electrode bands secured with Velcro straps around her waist, above both knees, and above both ankles. This configuration stimulated most of the lower limb muscles simultaneously between the belt electrodes [7]. The stimulation was carried out in “disuse mode” (stimulation frequency: 20 Hz; pulse width: 250 µs, duty cycle: five seconds of tetanic contraction followed by two seconds of rest) for muscle strengthening. The current intensity was always set to the maximum the patient could tolerate and was assessed every five minutes. 

In addition, as part of the nutritional management, she was instructed to drink one glass of a nutritional supplement (REHADAYS, Otsuka Pharmaceutical Factory, Inc., Tokushima, Japan) after breakfast at home daily for six months. REHADAYS is a supplement that contains leucine, an essential amino acid used for skeletal muscle synthesis. This supplement contains 160 kcal of energy, 11.0 g of protein, and 2300 mg of L-leucine in every 125 mL (https://www.otsukakj.jp/en/healthcare/medicalfoods/rehadays/). The primary outcome was the SMI, and the secondary outcomes were the SDAI, body mass index, body fat percentage, knee extensor muscle strength, and 6 m gait speed. Knee extensor muscle strength was measured using a handheld dynamometer (μTas F-1, Anima Co., Ltd., Tokyo, Japan) while the patient performed isometric knee joint extension movement at maximum effort for approximately five seconds in sitting position; the highest of four values (two from both sides) was recorded as the knee extension strength. These outcomes were assessed before and after six months of rehabilitation treatment. At the end of the six-month treatment period, the patient was re-assessed for sarcopenia and malnutrition.

Follow-up and outcomes

The patient’s drug treatment was continued unchanged during the six-month rehabilitation treatment period (172 days in total). The average frequency of TES using belt electrodes was 1.6 sessions per week (37 sessions in total). The intake rate of the nutritional supplement drink was 95.9 % (165/172 days); the reason for the lack of intake on seven days was that the patient forgot.

The changes in the current intensities every five minutes during the 37 TES using belt electrodes sessions were as follows: the current intensity was gradually increased, with median values of 2.2 mA in the thighs and 0.5 mA in the lower legs at five minutes after the treatment started, 2.5 mA in the thighs and 0.6 mA in the lower legs at 10 minutes, 2.6 mA in the thighs and 0.6 mA in the lower legs at 15 minutes, and 2.7 mA in the thighs and 0.6 mA in the lower legs at 20 minutes. The changes in subjective intensities of the lower limbs were as follows: the rating of perceived exertion remained at a median of 13 (somewhat hard) on the Borg scale [12], and the pain associated with the current intensity was minimal at a median of 1 to 2 on the numerical rating scale [13]. No joint-related adverse events were reported during the TES using belt electrodes period.

Table 1 shows the clinical outcomes before and after six months of rehabilitation treatment. Changes are presented as absolute changes and percentage improvements, with the latter calculated using the following formula: (After − Before) / Before × 100. The SDAI decreased from 5.1 points to 2.7 points (−2.4 points, −47.1 %), indicating a shift from low disease activity to clinical remission. The patient's muscle mass increased, primarily in the lower limbs: from 5.09 kg to 5.27 kg on the right (+0.18 kg, +3.5%) and from 4.97 kg to 5.09 kg on the left (+0.12 kg, +2.4 %). Accordingly, the SMI increased from 5.3 kg/m² to 5.5 kg/m² (+0.2 kg/m², +3.8 %). Knee extensor strength increased from 31.0 kgf to 34.5 kgf (+3.5 kgf, +11.3 %), and 6 m gait speed improved from 0.5 m/s to 1.0 m/s (+0.5 m/s, +100.0 %). After six months of treatment, reassessment showed that the patient no longer met the diagnostic criteria for sarcopenia or malnutrition.

**Table 1:** Clinical outcomes before and after six months of rehabilitation treatment SDAI, Simplified Disease Activity Index: SMI, skeletal muscle mass index: HAQ, Health Assessment Questionnaire. Before: before six months comprehensive rehabilitation treatment, After: after six months comprehensive rehabilitation treatment. Handgrip strength: The higher value from either hand was used regardless of dominance (left hand in this case). Change indicates the absolute change (percentage improvement) from before to after the treatment. The absolute change is calculated as *After − Before*, and the percentage improvement as *(After − Before) / Before × 100*.

	Before	After	Change
SDAI, point	5.1	2.7	−2.4 (−47.1)
Tender joint count	1	0	−1.0 (−100.0)
Swollen joint count	1	0	−1.0 (−100.0)
Evaluator global assessment, cm	1	1	0 (0)
Patient global assessment, cm	1.8	1.5	−0.3 (−16.7)
C-reactive protein, mg/dL	0.3	0	−0.3 (−100.0)
Body mass index, kg/m^2^	19.6	20	+0.4 (+2.0)
Body fat percentage, %	29.7	29.5	−0.2 (−0.7)
SMI, kg/m^2^	5.3	5.5	+0.2 (+3.8)
Hand grip strength, kg	22.1	22.9	+0.8 (+3.6)
Knee extensor muscle strength, kgf	31.0	34.5	+3.5 (+11.3)
6-m gait speed, m/second	0.5	1.0	+0.5 (+100.0)
HAQ scores	0.1	0	−0.1 (−100.0)

## Discussion

This case report is the first to provide evidence for the effectiveness of a rehabilitation treatment that combines wide-area, low-pain TES using belt electrodes and nutritional management in a patient with concurrent RA and sarcopenia. Our results suggest that a patient with concurrent RA and sarcopenia may be able to safely increase their skeletal muscle mass by receiving once- or twice-weekly TES using belt electrodes and daily nutritional management as rehabilitation treatment for six months.

Sarcopenia is characterized by selective atrophy of fiber size, mostly of fast-twitch muscle fibers [14]. Fast-twitch fibers follow the size principle and are recruited through strong muscle contractions [15]. This type of contraction requires moderate- to vigorous-intensity resistance training exceeding 60.0% of maximum voluntary contraction and is effective in increasing muscle mass and muscle strength. However, low-intensity resistance training is often chosen as rehabilitation treatment in the clinical settings because of the risk of worsening joint destruction in patients with RA. However, although low-intensity resistance training has good tolerance and treatment adherence, it is not sufficient to increase muscle mass and muscle strength [16]. To address this problem, this recently developed TES system using belt electrodes was adopted as an alternative to resistance training.

To ascertain the utility of TES using belt electrodes in the rehabilitation of patients with concurrent RA and sarcopenia, it is essential to consider not only its efficacy but also its overall safety profile and the continuity of its administration. In terms of efficacy, TES generally recruits both fast-twitch and slow-twitch muscle fibers, regardless of the current intensity. This makes it easier to recruit fast-twitch muscle fibers in TES than in resistance training [17]. However, TES bypasses the physiological size principle and activates motor units non-selectively, which may result in neuromotor incoordination. Despite these limitations, TES is a valuable option for patients who cannot perform high-intensity voluntary exercise, as it can recruit fast-twitch muscle fibers. On the other hand, conventional TES has limitations, such as a limited range of skeletal muscle coverage and the stimulation of sensory nerves in the skin, which occurs prior to the stimulation of muscles and causes pain. The TES applied in this case can induce stronger and more widespread muscle contractions with less pain during stimulation than conventional TES. Previous studies have shown that this method yields particularly positive results in patients who find it difficult to perform therapeutic exercise in a clinical setting such as a hospital [7]. Our patient reported that the stimulation during TES caused minimal pain. Furthermore, there was an increase in the muscle mass and muscle strength of the lower limbs after the 6-month rehabilitation treatment.

The stimulus intensity settings in the TES applied in this case were primarily based on the patient's subjective perceptions, with strong or maximum tolerance being considered appropriate in previous studies [7]. As the patient becomes habituated to the stimulation, the subjective intensity level gradually decreases within and between single TES sessions. For this reason, the set intensity within a single TES session was set to the maximum tolerable range at that time and re-evaluated every 5 minutes to gradually increase the intensity. The acceptable intensity level was also gradually increased between training sessions. In terms of the safety profile, TES using belt electrodes is likely to cause the following adverse effects: worsened disease activity, worsened joint symptoms, and cardiovascular-related complications. Regarding the disease activity, the SDAI in our case indicated a change from low disease activity to remission. Notably, the patient’s pharmacologic regimen remained unchanged during the intervention period. Evidence suggests that increasing physical activity and/or exercise simultaneously improves RA symptoms [18]. Therefore, the improvement in disease activity may have been partly attributable to the combined effects of TES and nutritional management, in the context of ongoing pharmacologic treatment. Overall, in the present case, there was at least no evidence of disease activity worsening. Regarding the joint symptoms, in contrast to active and passive exercises in conventional rehabilitation treatment, which entail joint movement and joint loads, TES involves the performance of isometric muscle contractions in a non-weight-bearing position, which theoretically decreases the risk of joint-related complications. In the present case, there were no joint-related adverse events during the TES period. The Borg scale was used to monitor the cardiovascular risks, and no serious cardiovascular events occurred. However, future research may require more careful monitoring methods.

At the start of the intervention, our patient exhibited malnutrition. Previous studies have reported that patients with RA have an adequate dietary intake and may develop malnutrition [19]. Appropriate nutritional management is considered a key strategy for maximizing the effects of resistance training on increases in muscle mass and strength [20]. Protein, in particular, is an essential dietary component of muscle synthesis and a well-known dietary recommendation for sarcopenia in the general population. Accordingly, our intervention included educating the patient on protein intake and incorporating a daily nutritional supplement throughout the 6-month rehabilitation period. The nutritional supplement used in this case contained a high amount of leucine, which is known to promote muscle protein synthesis. Adherence to the nutritional supplement regimen remained high throughout the 6-month rehabilitation period. This intervention not only improved her nutritional status but may also have enhanced the effects of TES treatment on increasing muscle mass and strength. However, the patient's overall dietary intake outside of the prescribed nutritional supplement was not assessed, which limits the ability to fully evaluate the contribution of nutritional management to the observed improvements. Given that many patients with RA typically receive only limited outpatient rehabilitation once or twice a week, daily nutritional management may be an important factor in supporting muscle maintenance and recovery in this population. Future studies should include dietary assessments, such as food diaries or dietary recall, to better isolate the effects of nutritional management.

Considering our findings, although some improvements in body composition, such as in BMI, body fat percentage, and SMI, were modest and of limited clinical relevance, the overall changes in disease activity, lower limb muscle strength, and gait speed suggest that the intervention contributed meaningfully to the patient's recovery from sarcopenia. These results indicate that the combined use of wide-area, low-pain TES and nutritional management may represent a feasible therapeutic option for patients with concurrent RA and sarcopenia. However, this case report has several limitations, including its single-case design, the absence of a control group, and the lack of long-term follow-up. Therefore, while the present findings are encouraging, they should be interpreted with caution. Further large-scale studies are warranted to validate these findings and assess their generalizability.

## Conclusions

In this case, although some of the improvements were modest, the combined intervention contributed to recovery in muscle function and nutritional status without any adverse events. This rehabilitation program may offer a feasible and safe therapeutic option for patients with concurrent RA and sarcopenia, particularly those who are unable to engage in resistance training. However, as this case involved early-stage rheumatoid arthritis with a low inflammatory burden, the findings may not be generalizable to all patients with RA. Further large-scale studies are warranted to evaluate the effectiveness and safety of this combined intervention in a broader population of patients with concurrent RA and sarcopenia. In particular, introducing comparative group designs (e.g., TES alone, resistance training alone, and their combination) may be beneficial for increasing the scientific rigor of future studies.
